# The elongation factor Spt5 facilitates transcription initiation for rapid induction of inflammatory-response genes

**DOI:** 10.1038/ncomms11547

**Published:** 2016-05-16

**Authors:** Gil Diamant, Anat Bahat, Rivka Dikstein

**Affiliations:** 1 Department of Biomolecular Sciences, The Weizmann Institute of Science, Rehovot 7600, Israel

## Abstract

A subset of inflammatory-response NF-κB target genes is activated immediately following pro-inflammatory signal. Here we followed the kinetics of primary transcript accumulation after NF-κB activation when the elongation factor Spt5 is knocked down. While elongation rate is unchanged, the transcript synthesis at the 5′-end and at the earliest time points is delayed and reduced, suggesting an unexpected role in early transcription. Investigating the underlying mechanism reveals that the induced TFIID–promoter association is practically abolished by Spt5 depletion. This effect is associated with a decrease in promoter-proximal H3K4me3 and H4K5Ac histone modifications that are differentially required for rapid transcriptional induction. In contrast, the displacement of TFIIE and Mediator, which occurs during promoter escape, is attenuated in the absence of Spt5. Our findings are consistent with a central role of Spt5 in maintenance of TFIID–promoter association and promoter escape to support rapid transcriptional induction and re-initiation of inflammatory-response genes.

## Abstract (web summary)

A subset of inflammatory-response NF-κB target genes is activated immediately after proinflammatory stimulation. Here, the authors show that in rapidly induced genes, depletion of the elongation factor Spt5 reduces transcription initiation and TFIID–promoter association without affecting the transcription elongation rate.

## Graphical abstract

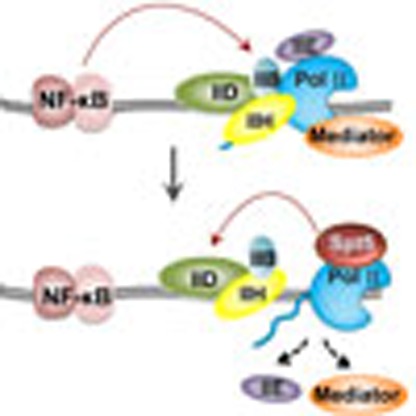

Eukaryotic transcription initiation of protein coding and non-coding genes occurs when RNA polymerase II (Pol II) together with general transcription factors are recruited to the promoter and form the pre-initiation complex (PIC). During the transition from initiation to elongation the Pol II breaks its interactions with the general transcription factors, escapes the promoter and enters into the elongation phase of RNA synthesis^1^,^2^,^3^.

The transcription elongation factor DRB sensitivity-inducing factor (DSIF), plays a central role in promoter-proximal pausing by Pol II (ref. 4). Spt5, the large subunit of DSIF, exhibits extensive homology to the NusG protein in eubacteria and archaea. In addition it has a long C-terminal domain, which bears a repetitive sequence called CTR. The CTR can be phosphorylated by P-TEFb, and this modification converts it from a negative to positive elongation factor^5^,^6^,^7^,^8^. In addition to proximal promoter pausing, DSIF was shown to interact with messenger RNA (mRNA) capping enzymes and facilitate capping^9^,^10^,^11^,^12^ and to coordinate elongation with mRNA splicing and export^13^,^14^. Recently DSIF was also reported to promote 3′ processing of snRNAs^15^ and DNA cleavage during immunoglobulin class switching^16^.

Nuclear factor (NF)-κB family of transcription factors is central to immune response as it induces the expression of major inflammatory genes as well as cell cycle and cell survival genes. In resting cells NF-κB is held in the cytoplasm in an inactive form by IκB proteins. Extra-cellular signals that activate NF-κB trigger degradation of IκBs resulting NF-κB nuclear translocation^17^,^18^. NF-κB activity is transient because two of its early target genes, *IκBα* and *A20*, are themselves negative regulators of NF-κB signalling. At the transcription elongation level most NF-κB target genes are controlled by the Brd4-P-TEFb pathway^19^,^20^,^21^,^22^,^23^,^24^,^25^,^26^,^27^,^28^,^29^,^30^,^31^. However a subset of NF-κB-response genes that includes the negative regulators *IκBα* and *A20*, are refractory to P-TEFb and Brd4 inhibition^20^,^27^,^29^,^32^. Upon their activation by NF-κB, DSIF occupancy of promoters proximal and coding regions is enhanced while P-TEFb is released and Pol II CTD remains hypo-phosphorylated^13^,^32^. In these genes DSIF has a major role in coordinating elongation with mRNA splicing and nuclear export^13^. In a recent global analysis it was found that the dependency of NF-κB target genes on Spt5 is linked to the rate of induction^14^.

The initial objective of the present study was to address the impact of Spt5 on the rate transcription elongation of inflammatory-response target genes. While elongation rate measurements did not reveal a defect in elongation speed, a striking and unexpected effect on transcript accumulation at the most 5′-end of the gene and the initial time points was observed, reminiscent of a defect in initiation or early elongation. This effect is not dependent on Spt4, the small subunit of DSIF and requires the NGN and KOW domains of Spt5, which are involved in Pol II interaction. Addressing the role that Spt5 plays in early stages of transcription revealed that it maintains threshold levels of the H3K4me3 and induced levels of H4K5Ac in the proximal promoter. Consequently the induced but not basal levels of TFIID–promoter association are abolished in the absence of Spt5. On the other hand levels of TFIIE are barely changed and that of Mediator are enhanced, suggesting that compared with TFIID, the association of these factors with the PIC is enhanced. Considering the recently emerging notion that displacement of both TFIIE and Mediator from Pol II is necessary for initiation to early elongation transition, these findings suggest that Spt5 has a role in promoter escape. Our findings are consistent with a central role of Spt5 in maintenance of TFIID–promoter association and promoter escape to support rapid transcriptional induction and re-initiation of inflammatory-response genes.

## Results

### Spt5 KD affects early transcription of NF-κB target genes

**Figure 1:**
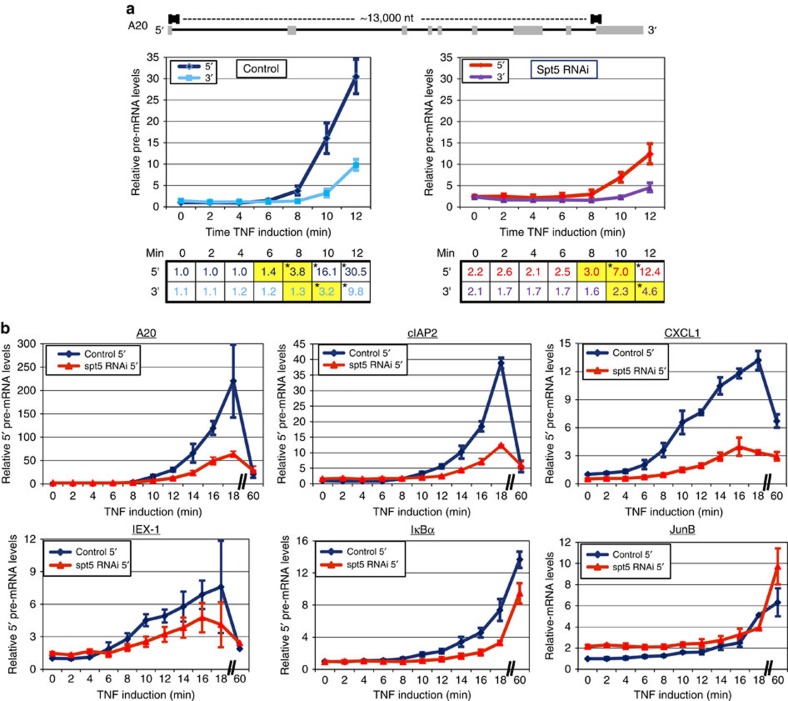
Kinetics of primary transcript accumulation of inflammatory-response genes. (**a**) Control and Spt5 KD cells were treated with TNFα for the indicated times and then total RNA was extracted and treated with DNaseI. Reverse transcription (RT) with random hexamers was followed by qPCR that included controls without reverse transcriptase and GAPDH for normalization. Schematic representation of the *A20* gene structure and the position of the primers used for the qPCR are shown on the top. The graph represents the induction rate in control (left) and Spt5 KD cells (right). Data are presented as mean±standard error of the mean (s.e.m.) of three independent experiments. The tables beneath each graph show the actual induction fold at each time point. The differences in the timing of induction between control and Spt5 KD samples are highlighted. Asterisk denotes statistical significant difference (*P*<0.05, *t*-test) compared to time 0. Downregulation of Spt5 was verified for each experiment by western blot. (**b**) Analysis of 5′end primary transcript accumulation (unless otherwise stated, first exon–intron junction) of the indicated genes as described in **a**. The graphs show the mean ±s.e.m. of the induction rate in control (blue) and Spt5 KD cells (red) of three independent experiments.

Induction of NF-κB by 1-h tumor necrosis factor (TNF)α treatment in Spt5 knockdown (KD) cells revealed substantial increase of pre-mRNA levels. In addition, a significant amount of processed mRNA is retained in the nucleus resulting in diminished protein accumulation^13^. To investigate underlying basis for the various mRNA processing defects exerted by Spt5 we hypothesized that Pol II elongation rate is linked to mRNA processing and that this rate is modulated by Spt5. To test this possibility we measured the elongation kinetics of a subset of NF-κB target genes. Control and Spt5 KD cells were induced with TNFα and harvested every 2 min. We confirmed that with our KD protocol the Spt5 occupancy of TNFα-induced genes is markedly diminished (Supplementary Fig. 1). We then monitored the induction of the primary transcript from the beginning (first exon–intron junction) and the end (last exon–intron junction) of these genes. These time points allow us to quantify the newly synthesized transcript before it is processed and other indirect effects accumulate. Figure 1a shows the data for the early time points of the *A20* gene. Induction of the 5′ end of the A20 gene in control cells is first apparent at about 6–8 min, and induction of the 3′ region of the transcripts at about 8–10 min (Fig. 1a, left). This 5′ to 3′ time interval suggests for an elongation rate of∼3.2 kb min^−1^ which is in line with the elongation rate measured for Pol II in other studies^33^,^34^. In Spt5 KD cells we observed several differences. First, at time 0 there are slightly higher initial transcript levels at both 5′ and 3′ ends (Fig. 1a, see time 0 in the tables beneath each graph), which is expected due to reduced proximal promoter pausing^35^,^36^. Second, induction at the 5′ is slightly delayed and first apparent at about 8–10 min and at 3′ at about 10–12 min (Fig. 1a, see highlighted numbers in the tables beneath each graph), suggesting that in Spt5 KD cells the time lapse between 5′ and 3′ is similar to control cells and elongation rate is actually comparable. Third, there is a significant and unexpected drop in the fold of induction at the 5′ end (Fig. 1a,b), which implies for a defect in early elongation or initiation. We therefore compared the effect of Spt5 KD on the kinetics of 5′-end transcript accumulation of several other NF-κB response genes. Similar to *A20*, the induction at the 5′end of *cIAP2*, *CXCL1* and *IEX-1* genes is substantially decreased following Spt5 KD (Fig. 1b). With the *IκBα* gene, we could not find suitable primers for detection of the 5′ primary transcript due to high GC content in this region; we therefore utilized primers from the first exon. Nonetheless, the induction fold at the 5′ is lower in the KD cells at the early time points as seen for the other genes (Fig. 1b). The induction of the intron-less *JunB* seems to be less affected by Spt5 KD.

After 1 h, levels of *A20*, *cIAP2*, *CXCL1* and *IEX-1* primary transcript drop as these are being spliced. Consequently this reduction is not evident in JunB and IκBα for which exonic primers were used. It should be noted that the effect observed at early time points are, by and large, not seen at later times (compare 1 h point in control and Spt5 KD samples). This can be explained by indirect effects, as following Spt5 KD the protein synthesis of A20 and IκBα, which are both negative regulators of NF-κB, is substantially reduced due to splicing and nuclear export defects (ref. 13 and Fig. 3b). Consequently the strength and the duration of NF-κB activity are markedly increased^13^. This failure to properly terminate NF-κB signalling in the absence of Spt5, leads to continuous synthesis of its target gene mRNA and the increase in their levels at later time points, in spite of Spt5 depletion.

In summary, we observed a striking effect of Spt5 KD on transcript induction levels at the very 5′ end and at the earliest time points suggesting an additional and unexpected role in controlling stages before elongation.

To elucidate the surprising activity of Spt5 at the earliest stages of transcription, we first considered the observation that Spt5 KD causes release of proximal promoter pausing of the *A20* gene so the basal state of A20 mRNA and protein rise slightly (∼twofold; Fig. 1a, see tables beneath the graphs). As A20 is an inhibitor of NF-κB signalling, we postulated that the higher basal levels of A20 (and potentially other negative feedback regulators) might be sufficient to cause a delay in the initial NF-κB response not detectable at later stages. This would translate into decreased activation folds in the early stages of induction. We therefore measured nuclear translocation of p65 in short time intervals immediately after TNFα induction. It is clear that nuclear translocation in not hindered but rather expedited in KD cells (Supplementary Fig. 2), indicating that this is not the cause of retarded induction when Spt5 is depleted. The enhancement of nuclear p65 may be the consequence of a slight reduction in basal IκBα levels or increased stability of nuclear p65. It is also interesting to note the temporal proximity of NF-κB nuclear translocation (Supplementary Fig. 2) and transcription activation (Fig. 1) which indicates that NF-κB finds its targets and activates them extremely fast.

### Spt5 NGN and KOW domains required for early transcription

**Figure 2:**
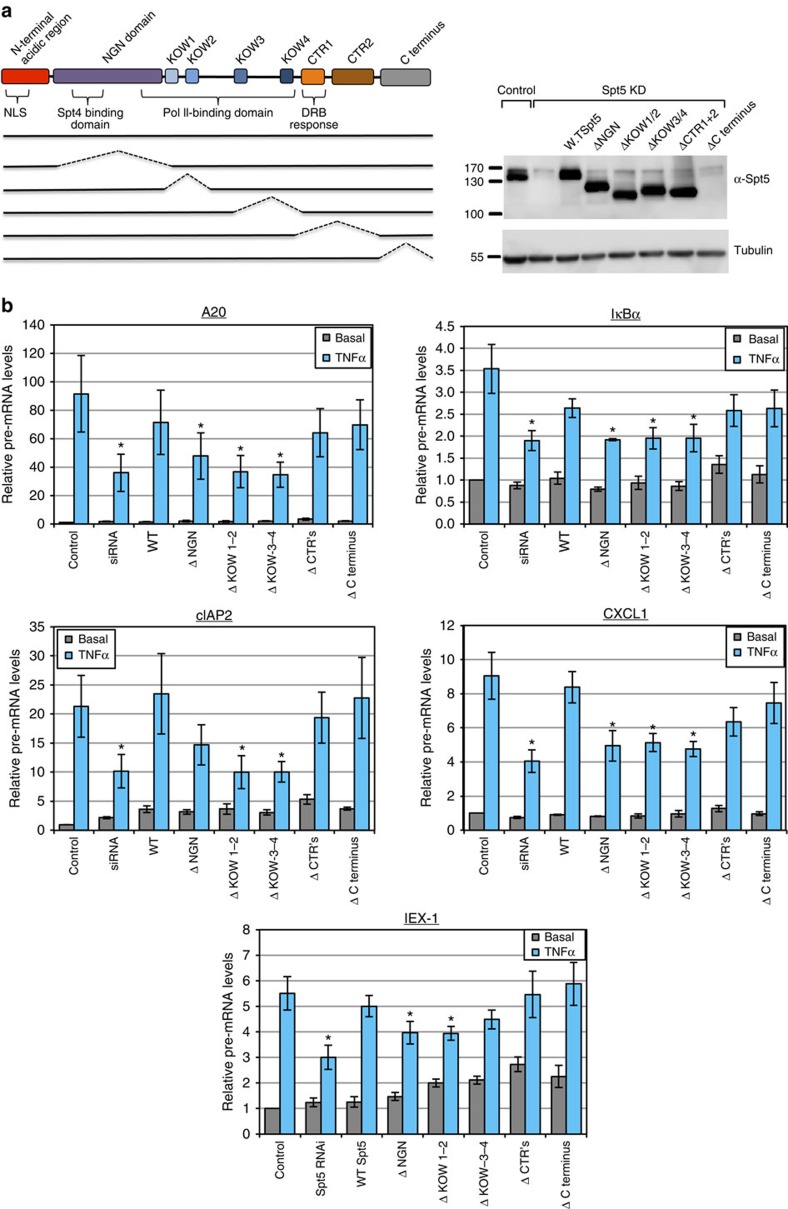
Mapping Spt5 domains important for the regulation of early transcription of NF-κB-induced genes. (**a**) A schematic representation of Spt5 domains and mutants are shown on the left. HeLa cells were transfected either with control and Spt5 RNAi or Spt5 RNAi together with expression plasmid of Spt5 protein variants. Expression of the various mutants was verified by western blot using Spt5 antibodies. As the antibody epitope is located at the most C-terminal part, the ΔC terminus mutant domain is not detected by it. The original images of the blots are shown in Supplementary Fig. 3. (**b**) Cells transfected as in A were induced by TNFα for 15 min followed by total RNA extraction, DNaseI treatment, reverse transcription and qPCR analysis using primers that detect only nascent 5′ transcripts of each gene. The graphs represent the mean ±s.e.m. of three independent experiments. Asterisk denotes statistical significant difference (*P*<0.05, *t*-test) compared with WT Spt5.

Spt5 consists of several functional domains that include: a NusG N-terminal homology (NGN) domain that harbours the interaction regions with Spt4 and Pol II; four KOW domains which also direct interaction with Pol II; a repetitive heptapeptide motif in the C-terminal region called CTR that is critical for the positive elongation activity of Spt5 (refs 8, 37, 38); and a C-terminal domain with an unknown function (Fig. 2a). To determine the domains important for the early transcriptional induction of inflammatory-response genes, we examined the ability of Spt5 mutants to restore it following depletion of endogenous Spt5. Control and KD cells were treated with TNFα for 15 min. This time point was selected since primary transcripts are readily detected while splicing is not yet apparent (Fig. 1). Expression of exogenous Spt5 variants was verified by immunoblot except for the ΔC terminus which contains the antibody epitope (Fig. 2a). The wild-type Spt5 partially or fully restored early induction of primary transcripts of *A20*, *IκBα*, *cIAP2*, *CXCL1* and *IEX-1* while the mutants in NGN, KOW1+2 and KOW2+4 domains failed to do so (Fig. 2b). On the other hand, the ΔCTR 1+2 and the ΔC terminus mutants restored the early transcriptional induction similar to the wild-type protein (Fig. 2b). These results suggest that the positive elongation activity associated with the CTR domain (as well as with the DRB-mediated repression) is not required for the early transcriptional effect.

**Figure 3:**
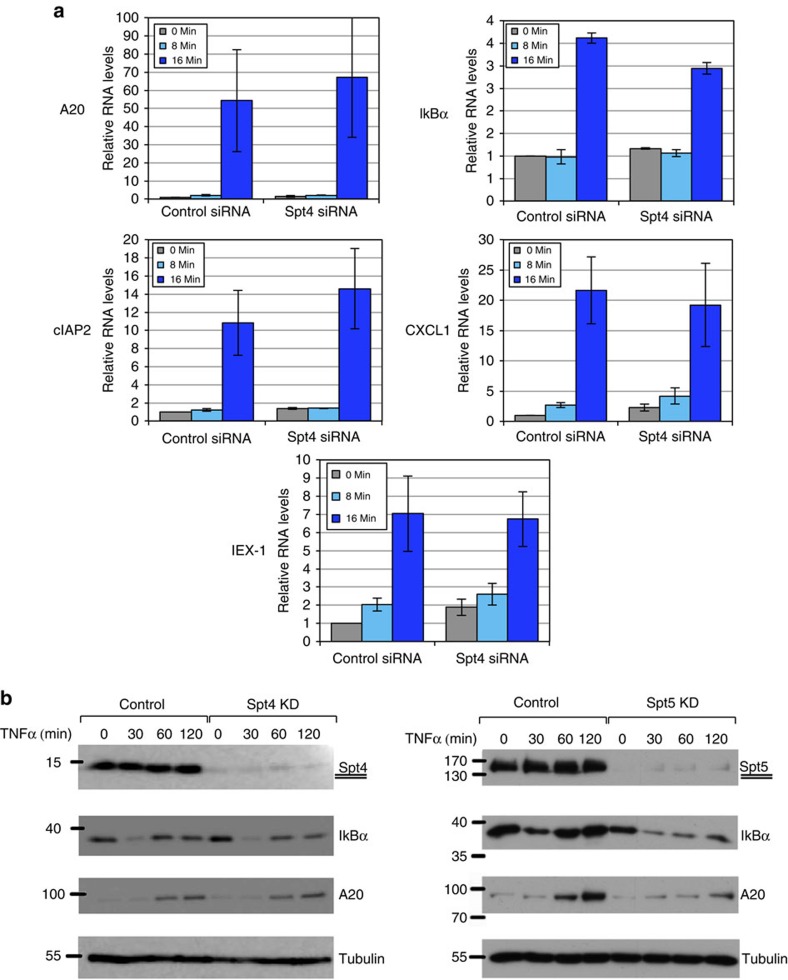
Effects of Spt4 and Spt5 on *A20* and *IκBα*. (**a**) Control and Spt4 KD cells were untreated or treated with TNFα for 8 or 16 min and then subjected to RT-qPCR analysis using primers that detect only nascent 5′ transcripts of the indicated genes. The graphs represent the mean±s.e.m. of three independent experiments. (**b**) Control and Spt4 KD cells (left) and Control and Spt5 KD cells were treated with TNFα for the indicated times. Levels of Spt4, Spt5, IκBα, A20 and β-Tubulin proteins were analysed by western blot.

To determine whether Spt4, the small subunit of DSIF, is required for this effect, Spt4 was downregulated by siRNA and the effect on the 5′ primary transcript induction was analysed 8 and 16 min following TNFα induction. The results revealed that efficient KD of Spt4 did not have any effect on early primary transcript accumulation (Fig. 3a). In addition, Spt4 was found to be dispensable for the induced protein levels of A20 and IκBα, while Spt5 is clearly essential (Fig. 3b) as previously reported^13^. These findings suggest that the middle domain of Spt5 involved in Pol II interaction but not the CTR involved in elongation facilitation, is the most important for the early transcription effect of Spt5.

### Spt5 regulates histone modifications of active promoters

**Figure 4:**
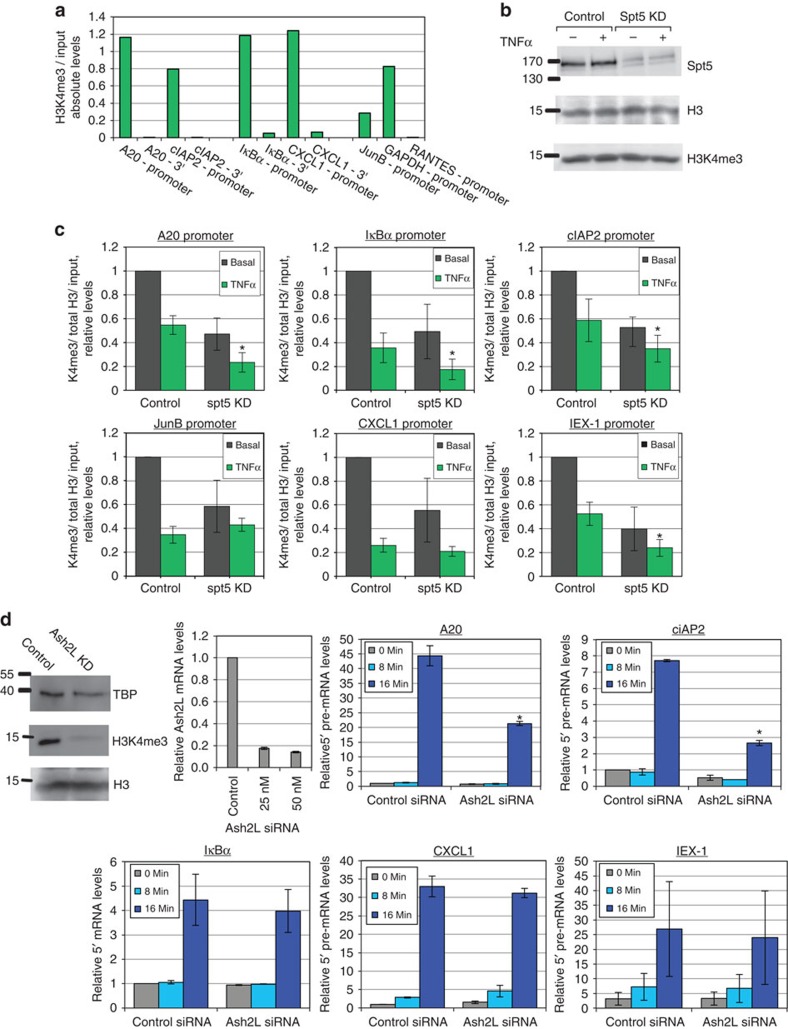
Spt5 KD lowers basal and induced levels of promoter-proximal H3K4me3 in NF-κB target genes. (**a**) ChIP was performed on untreated HeLa cells using H3K4me3. qPCR analysis was performed using primers from indicated loci of *A20*, *cIAP2*, *IκBα*, *CXCL1*, *JunB*, *GAPDH* and *RANTES* genes. (**b**) Control and Spt5 KD cells were treated with TNFα for 30 min or left untreated and protein levels of total histone H3 and H3K4me3 were analysed by western blot. The original images of the blots are shown in Supplementary Fig. 4. (**c**) Control and Spt5 KD cells were treated with TNFα for 30 min or left untreated, and then ChIP was performed using anti-H3K4me3 and anti-H3 antibodies. The 30 min time point chosen for the ChIP is within the peak of NF-κB and transcriptional activity. Analysis by qPCR was performed using primers from the proximal promoters of *A20*, *IκBα*, *cIAP2*, *JunB* and *CXCL1*. Graphs present levels of H3K4me3, normalized to H3 and relative to input, representing the mean±s.e.m. of 3–4 independent experiments. Asterisk denotes statistical significant difference (*P*<0.05, *t*-test) compared to the TNFα control. (**d**) Control and ASH2L KD cells were untreated or treated with TNFα for 8 and 16 min and then subjected to RT-qPCR analysis using primers that detect only nascent 5′ transcripts of the indicated genes. The graphs represent the mean±s.e.m. of 3 independent experiments. Asterisk denotes statistical significant difference (*P*<0.05, *t*-test) compared with the 16 min. TNFα control. Western blots showing the effect of ASH2L KD on the total H3, H3K4me3 and TBP (normalizing control) is shown on the left. The reduction of ASH2L expression following KD is validated by RT-qPCR and shown in the graph with the grey bars.

We next raised the possibility that Spt5 may affect transcription initiation rate by promoting specific histone modifications at the first nucleosome, which in turn facilitate recruitment of initiation factors. Such a modification is H3K4me3, which has been characterized as associated with proximal promoters of active genes^39^,^40^,^41^. A link between this modification and Spt5 was recently reported in immunoglobulin genes undergoing class-switch recombination^16^. We therefore tested whether Spt5 is required to maintain proximal promoter H3K4me3 of inflammatory-response genes. For this purpose, we performed chromatin immunoprecipitation assay (ChIP) using control and Spt5 knockdown cells with and without TNFα induction. We analysed levels of H3K4me3 and total H3 occupancy at the promoters of NF-κB target genes in which we recorded lowered initiation rates after knockdown. We first validated that the signal of this antibody is consistent with the known location of the modification (Fig. 4a). The results revealed that the basal level of H3K4me3 *A20* and *IκBα* genes is comparable to the level seen in the constitutively active gene *GAPDH*, and is restricted to the 5′end of the genes. As expected the signal from the *RANTES* gene (inactive in HeLa cells) is close to the background. These controls, along with several studies using the same antibody^39^,^42^ reassured us that we were detecting the correct modification. The relatively high levels H3K4me3 on *A20* and *IκBα* genes is consistent with the notion that these genes are ready for rapid transcriptional induction^43^. We validated that KD of Spt5 does not alter global levels of H3 or its K4me3 (Fig. 4b). Analysis of H3K4me3 following TNFα induction revealed that both total H3 and H3K4me3 levels were reduced in all genes, but the effect on H3K4me3 is more pronounced (Fig. 4c). This was initially surprising as we expected that TNFα-increased promoter activity would correlate with elevated H3K4me3 levels. However robust transcriptional induction as a consequence of frequent initiation is also expected to be associated with nucleosome dissociation/reorganization. Interestingly however, Spt5 KD decreased basal level of H3K4me3 in all genes examined and further lowered H3K4me3 levels following TNFα in *A20*, *IκBα* and *cIAP2*, whereas in *CXCL1* and *JunB* they were relatively unchanged (Fig. 4c).

To determine whether the H3K4me3 is important for the early stage of transcriptional induction, we knocked down ASH2L, a core subunit of the major H3K4 methyltransferase complexes MLL1-4, SET1A and SET1B. Downregulation of ASH2L was validated by RT-qPCR (Fig. 4d, left). This KD level of ASH2L was sufficient to cause a significant reduction in bulk H3K4me3 levels (Fig. 4d, left). We then analysed the effect of ASH2L depletion on the induction of the 5′ primary transcript of the pro-inflammatory genes and found that the levels of A20 and cIAP2 induced transcripts were reduced but those of IκBα, CXCL1 and IEX-1 were unchanged (Fig. 4d).

**Figure 5:**
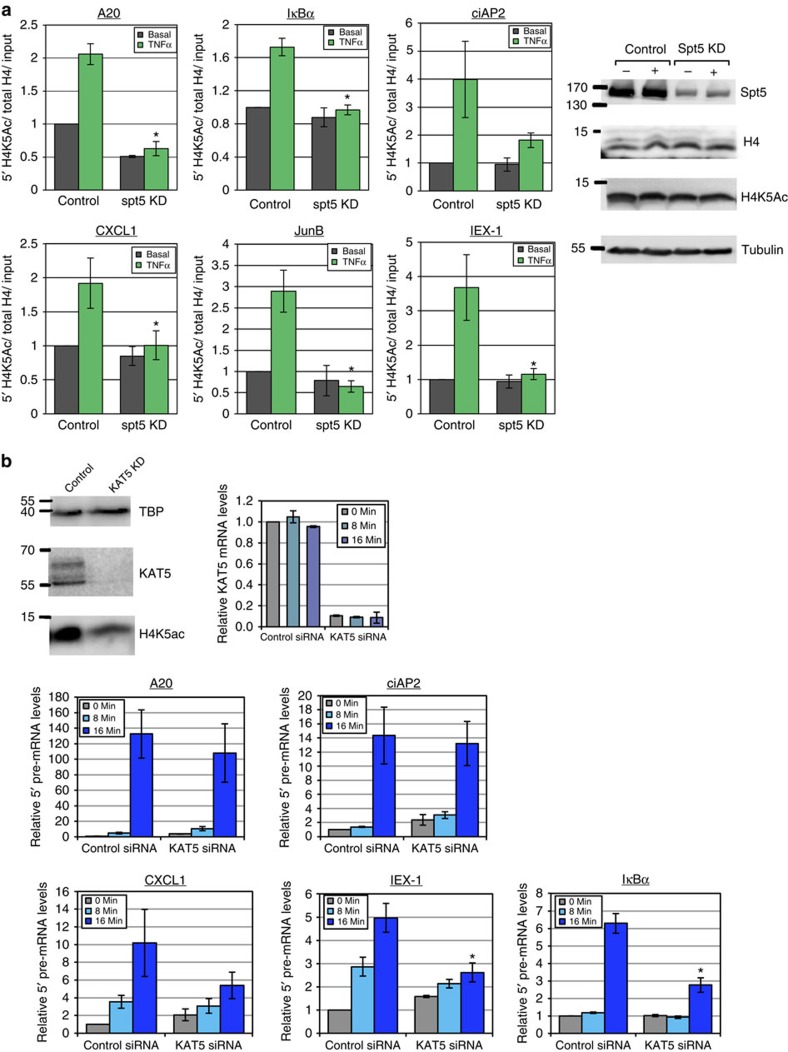
Spt5 KD diminishes induced levels of promoter-proximal H4K5Ac in NF-κB target genes. (**a**) Control and Spt5 KD cells were untreated or treated with TNFα for 30 min and then ChIP was performed using anti-H4K5A and anti-H4 antibodies. Analysis by qPCR was performed using primers from the proximal promoters of *A20*, *IκBα*, *cIAP2*, *JunB* and *CXCL1*. Graphs present levels of H4K5Ac, normalized to H3 and relative to input, representing the mean±s.e.m. of 3–4 independent experiments. Asterisk denotes statistical significant difference (*P*<0.05, *t*-test) compared to TNFα control. Western blot analysis of control and Spt5 KD cells, untreated or treated with TNFα for 30 min, using antibodies against total histone H4 and H4K5Ac, is shown on the right. (**b**) Control and KAT5 KD cells were untreated or treated with TNFα for 8 and 16 min and then subjected to RT-qPCR analysis using primers that detect only nascent 5′ transcripts of the indicated genes. The graphs represent the mean±s.e.m. of three independent experiments. Asterisk denotes statistical significant difference (*P*<0.05, *t*-test) compared to 16 min. TNFα control. Western blots showing the KD of KAT5 and its effect on the total H4, H4K5Ac and TBP (normalizing control) is shown on the top left.

As the effect of reduced H3K4me3 can only partially account for the observed effect on early transcription we examined another modification associated with the promoters of active genes. It has been established that acetylation of several residues of histone H4 N-terminal tail, in particular lysine 5, 8 and 12 are associated with active promoters^44^,^45^,^46^. To examine the potential role of Spt5 in maintaining/promoting H4 acetylation we analysed the acetylation of lysine 5 of H4 (H4K5Ac). Analysis of this modification of pro-inflammatory genes following TNFα induction using ChIP revealed its induction in all the analysed genes. Furthermore, the induced level of this modification was completely diminished by Spt5 KD in all tested genes (Fig. 5a). Spt5 depletion did not cause reduction in the global H4 and H4K5Ac (Fig. 5a, right), suggesting that its depletion prevented the induction of this modification specifically on the induced genes.

We next determined the functional significance of this modification for early transcription by examining KAT5 (Tip60), a histone acetyl transferase of H4K5 (refs 47, 48). KD of KAT5 by siRNA (Fig. 5b, left) resulted in global reduction of H4K5Ac. We then analyse the impact of diminished K4K5Ac on the early transcriptional induction of pro-inflammatory genes. Diminished K4K5Ac was associated with reduced activation of the primary transcript at early time point of *IκBα*, *CXCL1* and *IEX-1* but not *A20* and *cIAP2* (Fig. 5b, bottom). Interestingly, this effect is exactly the opposite than that of ASH2L KD and reduced H3K4me3. Thus Spt5 affects both modifications but the requirement of each is differential.

### Spt5 KD diminishes TNFα-induced recruitment of TFIID

**Figure 6:**
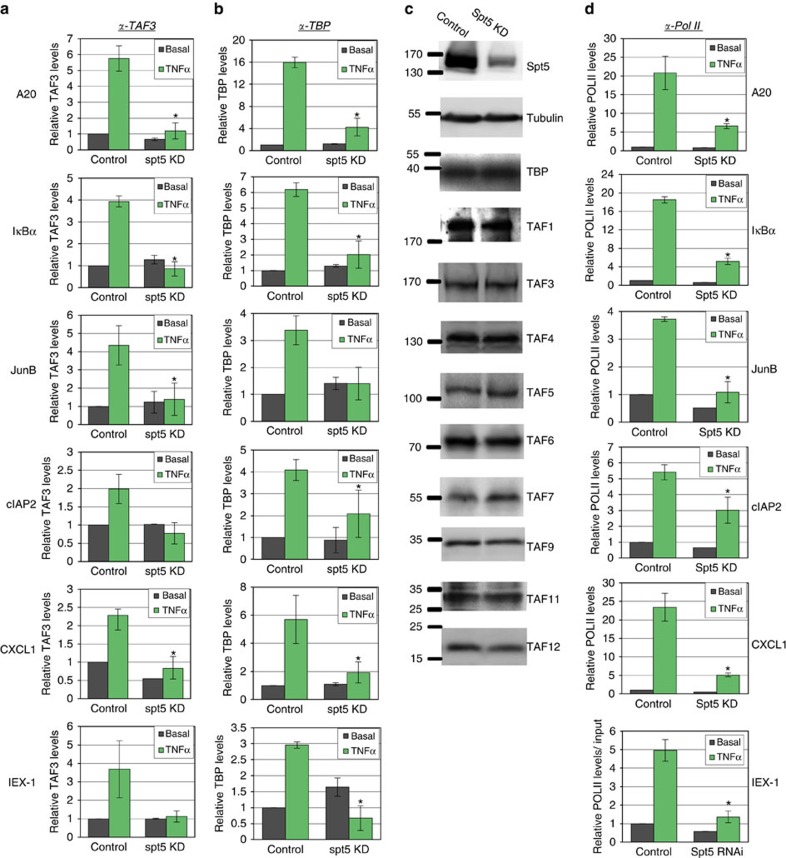
Spt5 KD diminishes TNFα-induced recruitment of TFIID. (**a**) Control and Spt5 KD cells were treated with TNFα for 30 min and then subjected to ChIP using anti-TAF3. Analysis by qPCR was performed using primers from promoters of *A20*, *IκBα*, *cIAP2*, *JunB* and *CXCL1*. Graphs represent levels relative to input (mean±s.e.m.) of 3–4 independent experiments. Asterisk denotes statistical significant difference (*P*<0.05, *t*-test) compared with TNFα control. (**b**) A ChIP assay as in A using anti-TBP antibodies. (**c**) Control and Spt5 KD cells were analysed by western blot with antibodies against Spt5, β-Tubulin, TBP and the indicated TAFs. (**d**) A ChIP assay as in A using anti-Rbp1 (major Pol II subunit) antibodies.

H3K4me3 and K4K5Ac modifications support maintenance of the general transcription factor TFIID on the promoter through direct interaction with the TAF3 and TAF1 subunits, respectively^39^,^40^,^41^,^49^,^50^. This raises the possibility that the reduction of H3K4me3 and K4K5Ac in NF-κB target genes upon Spt5 KD may hinder TFIID recruitment. To examine this idea we determined the effect of Spt5 KD on promoter occupancy by TBP, a core subunit of TFIID, and TAF3 a TFIID-specific subunit, using ChIP assays. TAF3 occupancy at the basal level was almost unchanged by Spt5 KD, indicating that Spt5 is not required for the basal occupancy levels of TAF3 (Fig. 6a). However, after TNFα induction, reduced Spt5 levels resulted in complete loss of TAF3-induced recruitment (Fig. 6a). Similar findings were observed for TBP, showing no reduction or even slight increase in basal occupancy, with considerable decrease in recruitment following induction (Fig. 6b). We then examined whether Spt5 KD affects the protein levels of TFIID subunits and found these to be unchanged (Fig. 6c). We also examined the effect of Spt5 KD on the levels of Pol II and found a substantial reduction in the induced occupancy at all promoters analysed (Fig. 6d). These results suggest a link between Spt5 KD and lowered initiation rates, TFIID and Pol II occupancies and altered H3K4me3 and H4K5Ac patterns. This link appears to be a fundamental part of Spt5′s regulation of activated inflammatory genes.

### Spt5 promotes Mediator and TFIIE release

**Figure 7:**
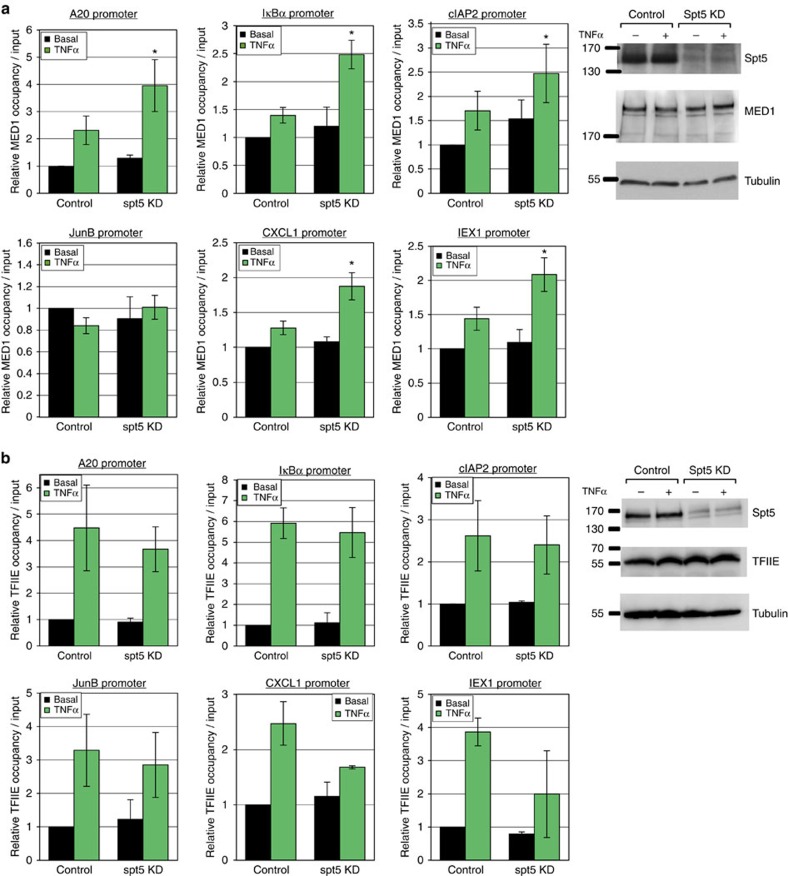
TFIIE and Mediator levels are not correlated with those of TFIID following Spt5 KD and TNFα induction. Control and Spt5 KD cells were treated with TNFα for 30 min and then subjected to ChIP using either anti-MED1 (**a**) or anti-TFIIEα (**b**). Analysis by qPCR was performed using primers from promoters of *A20*, *IκBα*, *cIAP2*, *JunB* and *CXCL1*. Graphs represent levels relative to input (mean±s.e.m.) of 3–4 independent experiments. Asterisk denotes statistical significant difference (*P*<0.05, *t*-test) compared to TNFα control. In each section a control western blot analysis of control and Spt5 KD cell lysates with the indicated antibodies is shown on the right.

To investigate further the mechanism by which Spt5 affects early transcription we raised the possibility that Spt5 may act at the transition from initiation to elongation. Recent studies in yeast showed that the Mediator is dynamically associated with the PIC and is rapidly dissociated during promoter escape. When promoter escape is impaired due to Kin28 (Cdk7) depletion, enhanced accumulation of the Mediator on the core promoter was found^51^,^52^. We therefore envisaged that if Spt5 is involved in promoter escape, a defect in Mediator complex displacement is expected upon its KD. To test this we examined Mediator occupancy by ChIP using antibodies against its Med1 (TRAP220) subunit. The results revealed modest but statistically significant increase of the Mediator levels on the promoters of the *A20*, *IκBα*, *cIAP2*, *IEX-1*, *CXCL1* but not *JunB* genes (Fig. 7a). Considering the sharp reduction in Pol II and TFIID levels upon Spt5 KD and TNFα induction, the Mediator to TFIID/Pol II ratio appears to be substantially elevated. These results suggest that Mediator dissociation from the PIC is impaired in the absence of Spt5.

To gain additional support for the involvement of Spt5 in promoter escape we also considered the *in vitro* studies with archea Spt5 which suggest that it competes with the initiation factor TFE (TFIIE homologue) for binding to Pol II (ref. 53) and another study showing the release of TFIIE during initiation to elongation transition^54^. To examine whether Spt5 KD interferes with TFIIE displacement from the PIC we analysed TFIIE occupancy using ChIP and found that TFIIE levels are by and large, barely changed (Fig. 7b). According to the stoichiometry of the PIC components we would expect the levels of TFIIE to drop as seen in TFIID and Pol II. However, it appears that TNFα induction in the absence of Spt5 actually increases the TFIIE to TFIID/Pol II ratio in all the analysed genes. We interpret this finding as an indication of a defect in TFIIE release.

## Discussion

**Figure 8:**
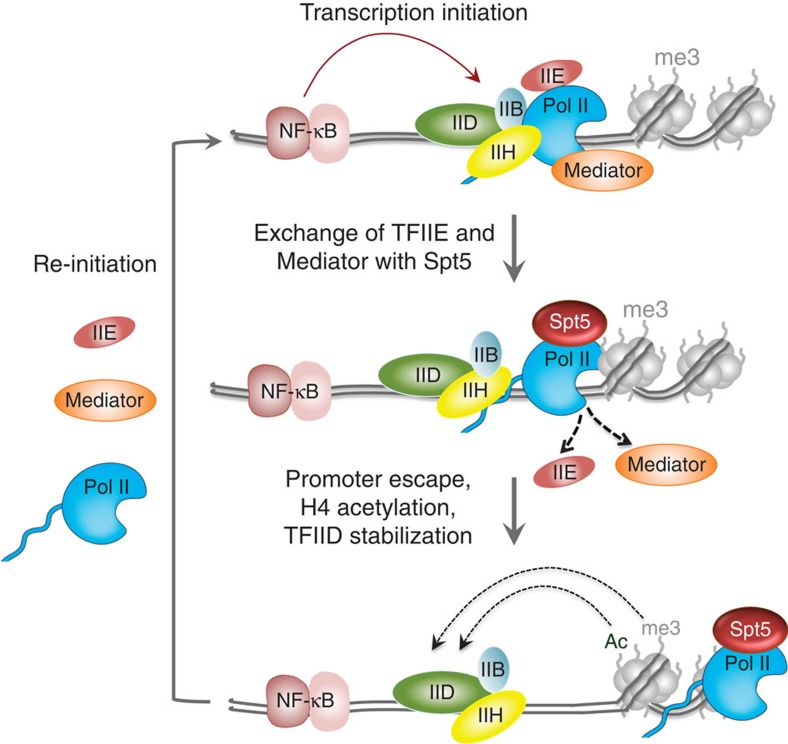
The role of Spt5 in early elongation. A model of the rapid transcriptional induction of inflammatory genes and the role of Spt5 in early elongation as described in the discussion.

DSIF is well known for its role in transcription elongation and RNA processing. In the present study we have uncovered a novel function of the Spt5 subunit of DSIF in supporting transcription re-initiation rates of NF-κB target genes. This conclusion is based on several observations. We found, for the first time, that Spt5 promotes histone H4 acetylation and maintains promoter-associated H3K4me3. In addition Spt5 supports TFIID recruitment and stabilization on activated promoters while facilitating Mediator and TFIIE release from Pol II. Our results suggest a model (Fig. 8) in which Spt5 is involved in displacing TFIIE and Mediator from the PIC during promoter escape. Successful promoter clearance is followed by transcription of the DNA spanning the first nucleosome, which triggers histone modifications that hold TFIID on the promoter. Stabilization of the TFIID–promoter interaction is important only when re-initiation is induced by NF-κB^43^,^55^ but not in resting state where the rate of promoter firing is low. The model is based on the observations that in the absence of Spt5, the TFIIE and Mediator dissociation from Pol II is impaired, promoter-proximal histone marks are reduced and TFIID–promoter interaction is destabilized.

By measuring the accumulation of 5′end pre-mRNA at the earliest time points after TNFα induction we observed retarded and diminished induction rates following Spt5 KD. We ruled out retarded kinetics of NF-κB activation and reduced Pol II elongation rate. Of all the possible mechanisms we examined, the most striking finding is a critical reduction in TFIID recruitment to TNFα-induced promoters upon Spt5 depletion, without any effect on TFIID subunits protein levels. Our findings reveal the involvement of Spt5 in facilitation of promoter-proximal histone modification, specifically H3K4me3 and H4K5Ac, known to stabilize TFIID association with promoters^39^,^40^,^41^,^49^,^50^. Furthermore, we addressed the functional significance of H3K4me3 and H4K5Ac marks, and observed a clear differential requirement. Induction of *A20* and *cIAP2* was dependent on H3K4me3 but not H4K5Ac, while the exact opposite was found for *IκBα*, *CXCL1* and *IEX-1*. Although we cannot rule out the possibility that the marks might be preferentially retained at the set of gene that were not affected by the KD, these findings fit the reported data in which the dependence on H3K4me3 for TFIID recruitment changes from gene to gene, where high affinity of promoter to TFIID through a TATA-box renders the genes less dependent on H3K4me3 (ref. 39). Our findings are in agreement with this notion as *A20* and *cIAP2* are both TATA-less while *IκBα*, *CXCL1* and *IEX-1* are driven by TATA or TATA-like promoters. It is therefore also possible that H4K5Ac is linked to TATA-containing promoter.

The positive effect of Spt5 on early transcription is dependent on the central and highly conserved domain harbouring the NGN and KOW domains. These domains are found in the archeal and bacterial homologues and were shown to promote elongation through interacting with RNA polymerase II and stabilizing its closed conformation^56^. This suggests that Spt5′s facilitation of initiation may be related to its ancient ability to interact with and stabilize the closed Pol II–DNA complex. On the other hand, the CTR and the C-terminal domains of Spt5 that are involved in its positive effect on elongation, as well as its Spt4 partner, are dispensable for this activity. This is in line with the work showing that phosphorylation of the CTR domains by P-TEFb after initiation is how these domains promote Pol II elongation^8^,^37^,^38^. The apparent lack of a defect in elongation rate can be explained by functional redundancy with other elongation factors such as TFIIF, which becomes engaged with the elongating Pol II at higher levels in the absence of Spt5 (ref. 13).

Another characteristic of promoter escape and entrance to the elongation phase is a rapid dissociation of Pol II from the general transcription factors. As mentioned above, the archeal Spt5 competes with TFIIE for *in vitro* binding of Pol II and is capable of displacing it^53^. Consistent with that in mammalian cells CDK7, the kinase subunit of TFIIH, was shown to promote release of TFIIE and recruitment of Spt5 (ref. 54). Also, Kin28, the yeast homologue of CDK7, was shown to facilitate the release of the Mediator complex. Its absence was associated with markedly elevated Mediator association with the core promoter, which was interpreted as a defect in promoter escape^51^,^52^. Considering these links between promoter escape and the release of both TFIIE and Mediator, the finding that Spt5 KD prevented displacement of both TFIIE and Mediator strongly support the idea that the initiation-to-elongation transition is impaired.

In summary our results uncover a previously unknown role of Spt5 in supporting multiple rounds of transcription, a hallmark of the rapid and efficient induction characteristic of inflammatory-response genes.

## Methods

### Cell culture and transfections

HeLa cells, originally obtained from ATCC, were grown and maintained in Dulbecco's modified Eagle's medium supplemented with 10% fetal calf serum (Gibco) and 1% penicillin-streptomycin. To avoid basal NF-κB activity, cells were kept from reaching confluence, and re-plated after initial thawing no more than 10 times. To knockdown Spt5 1,250,000 cells were plated in 10 cm dish and transfected 24 h later using ICAFectin441 (In-Cell-Art) and 12 μg or either pSUPER or a mix of two distinct pSUPER-spt5-RNAi, together with 0.5 μg CMV-GFP-Puro plasmid. Transfected cells were selected for 24 h with puromycin (1 μg ml^−1^) and then induced with 20 ng μl^−1^ TNF-α for the indicated times. For the Spt5 knockdown rescue by deletion mutants, 150,000 HeLa cells were transfected in 6-well plates with 3 μg DNA mix containing pSUPER, spt5-RNAi, or a combination of RNAi with the pLenti vector expressing wild-type or mutant Spt5. siRNAs against Spt4, ASH2L and KAT5 are from Dharmacon siGENOME SMART pool. Transfection of siRNA into HeLa cells was done using DharmaFECT1 transfection reagent (Dharmacon). The Dharmacon ON-TARGETplus Non-targeting siRNA #3 was used as a negative control.

### RNA preparation

Total RNA was extracted using Tri-reagent (MRC) according to manufacturer's instructions. The RNA was treated with DNase using the DNA free Turbo DNase kit (Ambion). RNA was ran on 1% agarose gel and imaged with ethidium bromide to validate sample quality. cDNA was prepared from 1 μg RNA using the *ABI* high capacity cDNA RT kit, and analysed by qPCR using the ABI 7,300 Real Time PCR system and the Power SYBR reaction mix for PCR (*ABI*).

### Chromatin Immunoprecipitation

HeLa cells (∼1.5 × 10^6^ per IP) were treated or left untreated with 20 ng ml^−1^ TNFα for 30 min and then cross-linked with 1% formaldehyde for 10 min at room temperature. Chromatin was then extracted and used for immunoprecipitation with the indicated antibodies. The immunoprecipitated DNA samples were purified using Qiagen PCR product purification columns and analysed by qPCR.

### Western blot and antibodies

Whole cell extract was prepared by lysing cells in 50 mM Tris pH8, 250 mM NaCl, 5 mM EDTA, 0.5% NP-40 and 1% protease inhibitor cocktail. Protein concentration of samples was measured by Bradford assay and equalized. Samples were then separated by SDS–PAGE and subjected to western blot. Antibodies against Spt5, TBP and several of the TAFs were previously described^13^,^43^,^57^. Spt4 antibody is a kind gift of Dr Yuki Yamaguchi (Tokyo Institute of Technology). KAT5 antibody is from Antibody Verify (#AAS92387C); H3 (#Ab-1791), H3K4me3 (#Ab-8580), Pol II (Ab-817) and TFIIEα (#Ab-28177) antibodies are from Abcam; H4 (#MM07-108), H4K5Ac (#MM07-327) and TAF3 (#MM07-1802) antibodies are from Millipore; Med1 (sc-8998), TAF1 (sc-735) and p65 (sc-8008) antibodies are from SantaCruz; IκBα (#610690) antibody is from BD-transductions; A20 (#60-6629-82) antibody is from eBiosciences. The uncropped blots are shown in Supplementary Figs 3–7.

### Data availability

All relevant data are included with the manuscript (as figure source data or Supplementary Information files). Additional information can be obtained from the corresponding author (R.D).

## Additional information

**How to cite this article:** Diamant, G. *et al*. The elongation factor Spt5 facilitates transcription initiation for rapid induction of inflammatory-response genes. *Nat. Commun.* 7:11547 doi: 10.1038/ncomms11547 (2016).

## Supplementary Material

Supplementary Information Supplementary Figures 1-7
