# Supplementary material for: The elongation factor Spt5 facilitates transcription initiation for rapid induction of inflammatory-response genes
Source: Nat Commun. 2016 May 16;7:11547. doi: 10.1038/ncomms11547 (PMC4873663; doi:10.1038/ncomms11547)
Supplement: Supplementary Information — Supplementary Figures 1-7 [file ncomms11547-s1.pdf]

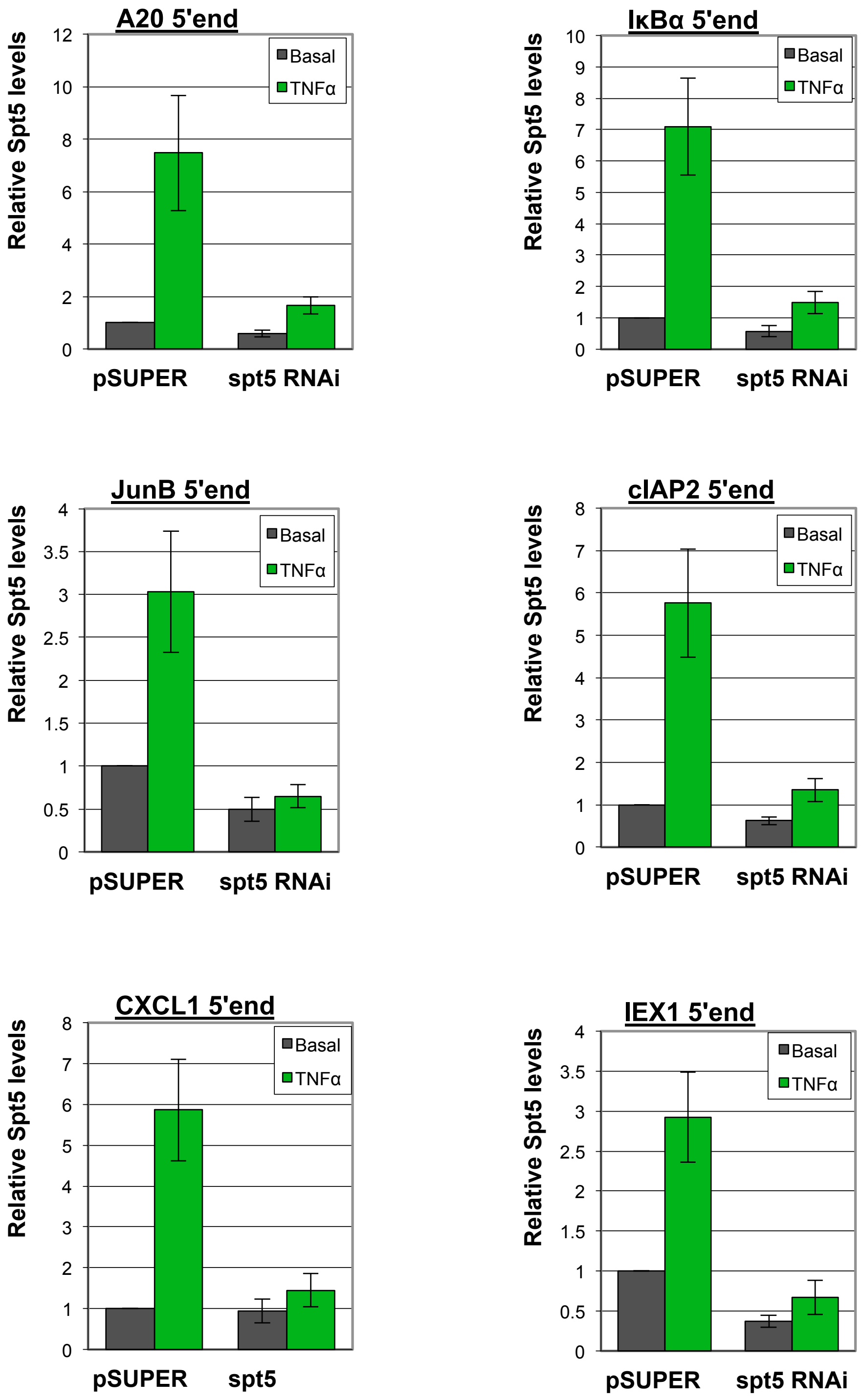

Supplementary Figure 1: Control and Spt5 KD cells were treated with TNFα for 30 minutes or left untreated, and then ChIP was performed using anti-Spt5 antibodies. The 30 min time point chosen for the ChIP is within the peak of NF-κB and transcriptional activity. Analysis by qPCR was performed using primers from the proximal promoters of A20, IκBα, cIAP2, JunB, CXCL1, and IEX-1. Graphs present Spt5 levels to input, representing the mean ± SEM of 4 independent experiments.

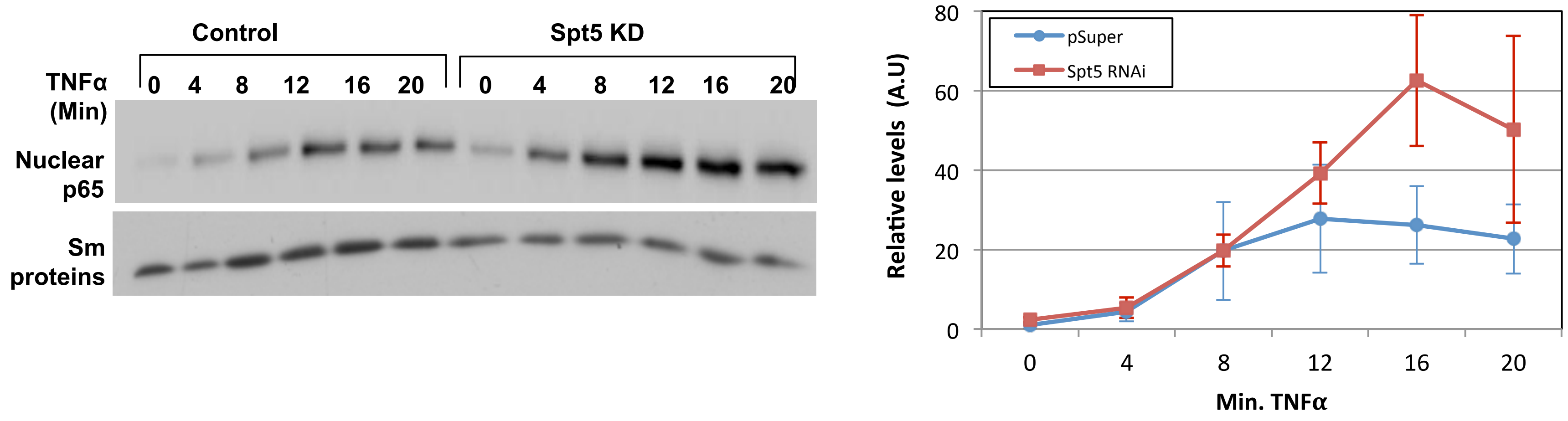

Supplementary Figure 2: Effect of Spt5 knockdown on rate of NF-κB nuclear translocation. Spt5 knockdown and control HeLa cells were treated with TNFα for 4, 8, 12, 16 and 20 minutes. Cells were then promptly harvested and protein extracted from the nuclear fraction. Levels of NF-κB subunit p65 were analyzed in each fraction by western blot and quantified. The graphs present quantified results of p65 levels normalized to levels of Sm proteins or TBP (abundant nuclear proteins, both validated as unresponsive to Spt5 depletion) levels from 3 independent experiments (mean ± SEM). The original image is shown in Supplementary Figure 3.

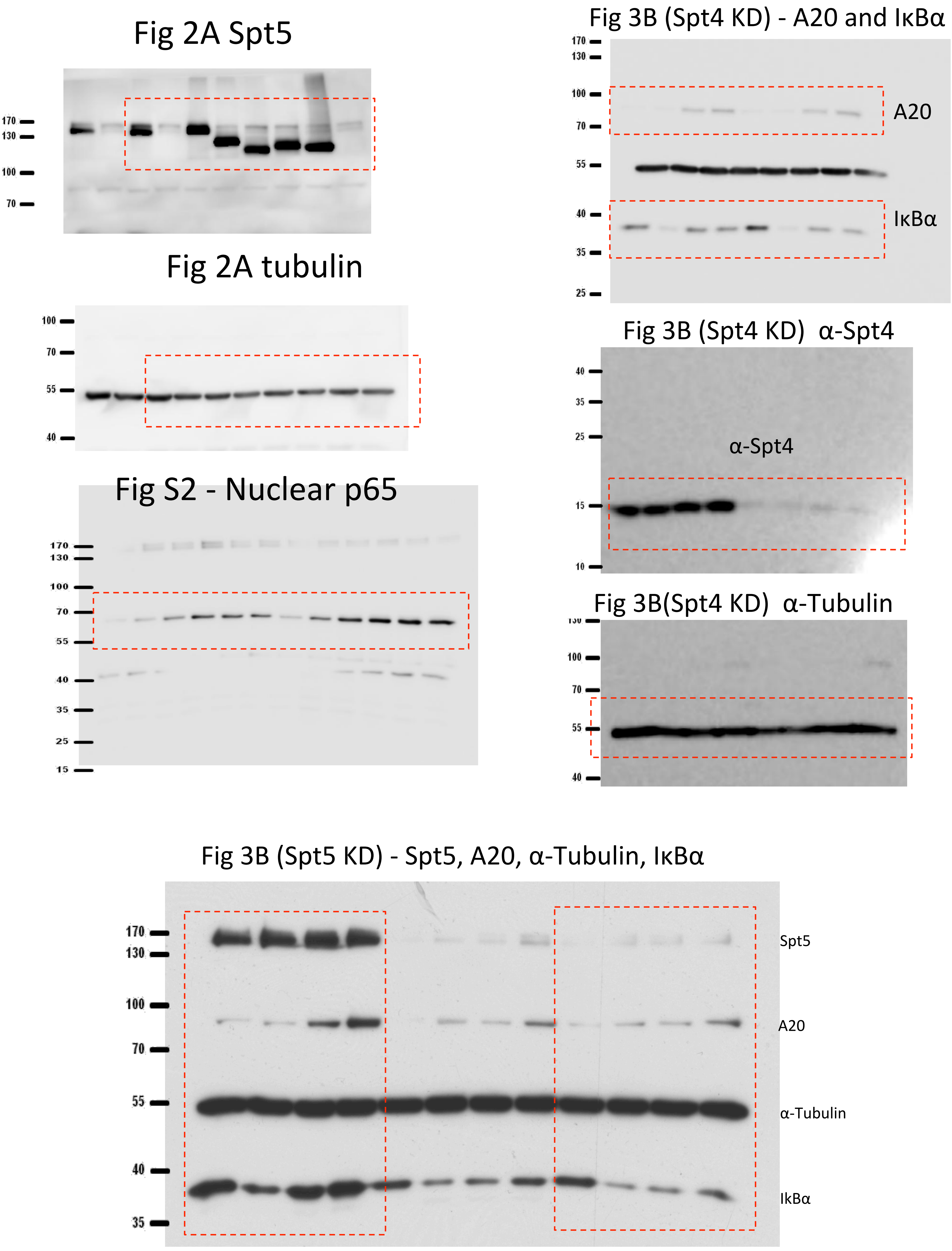

Supplementary Figure 3: Original images of western blots used for preparing figures 2A, 3B, and S2 .

Fig 4A – Spt5

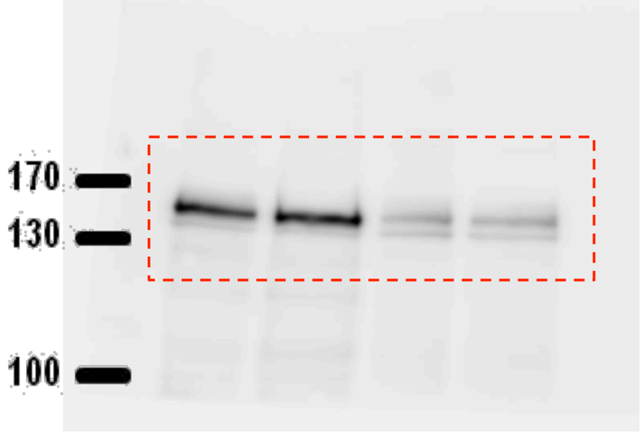

Fig 4A – H3

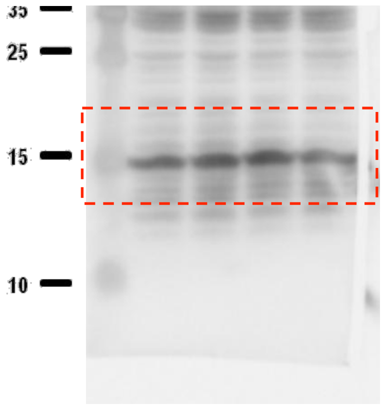

Fig 4A - H3K4me3

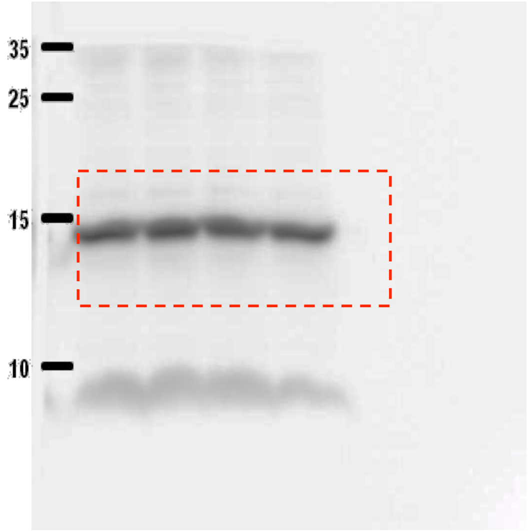

Fig 4C - TBP (top), H3K4me3 (bottom)

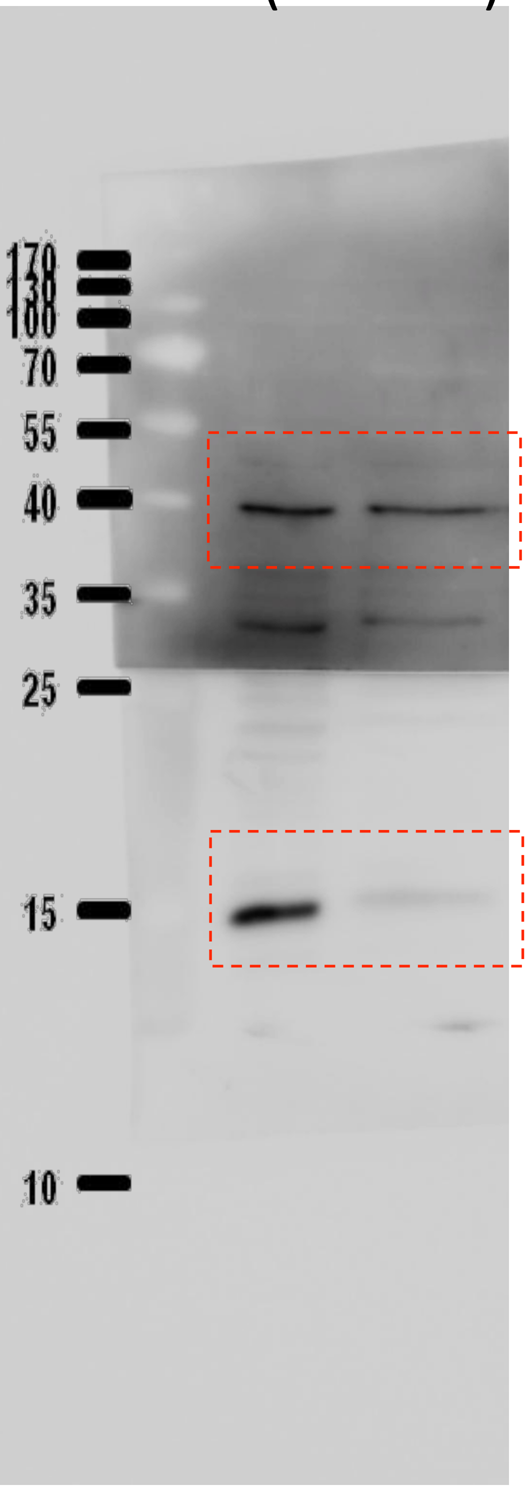

Fig 4C – H3

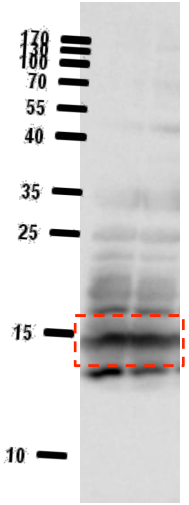

Supplementary Figure 4: Original images of western blots used for preparing figure 4.

Fig 5A – Spt5

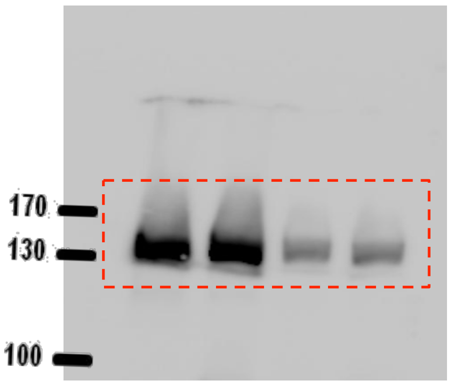

Fig 5A – H4

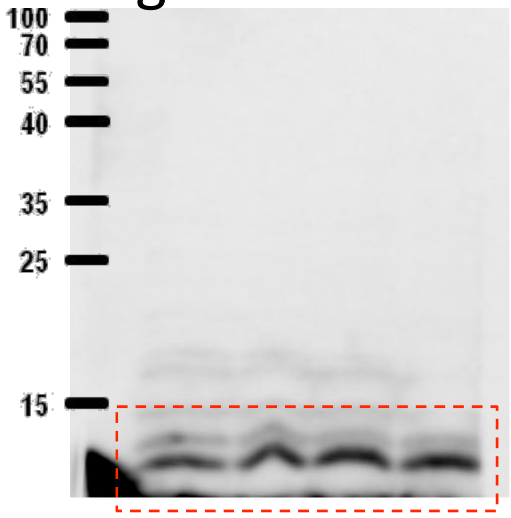

Fig 5A - H4K5ac

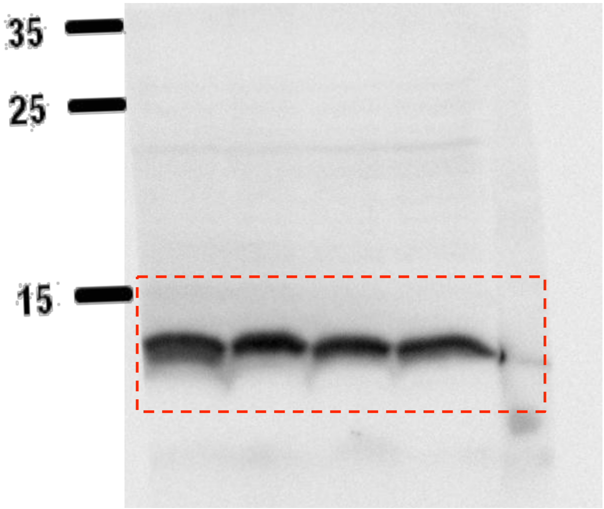

Fig 5A - Tubulin

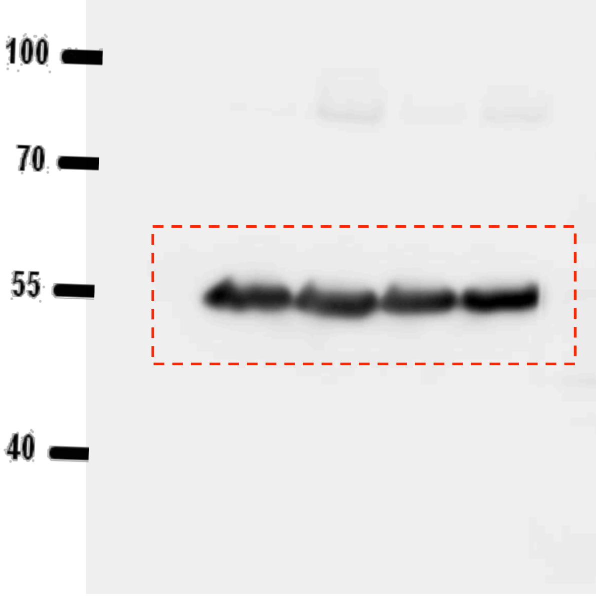

Fig 5B – KAT5

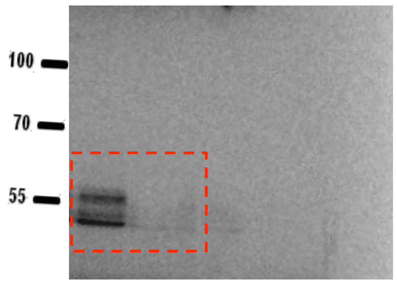

Fig 5B - H4K5ac

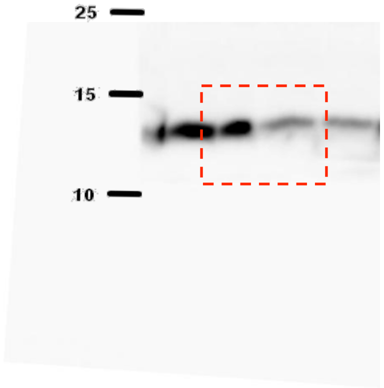

Fig 5B - TBP

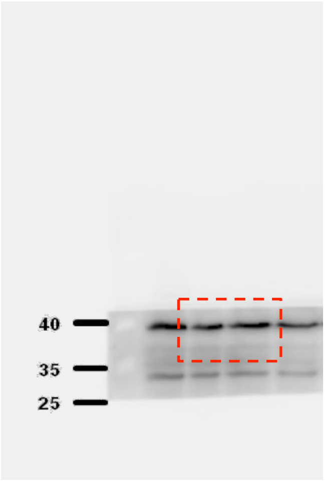

Supplementary Figure 5: Original images of western blots used for preparing figure 5.

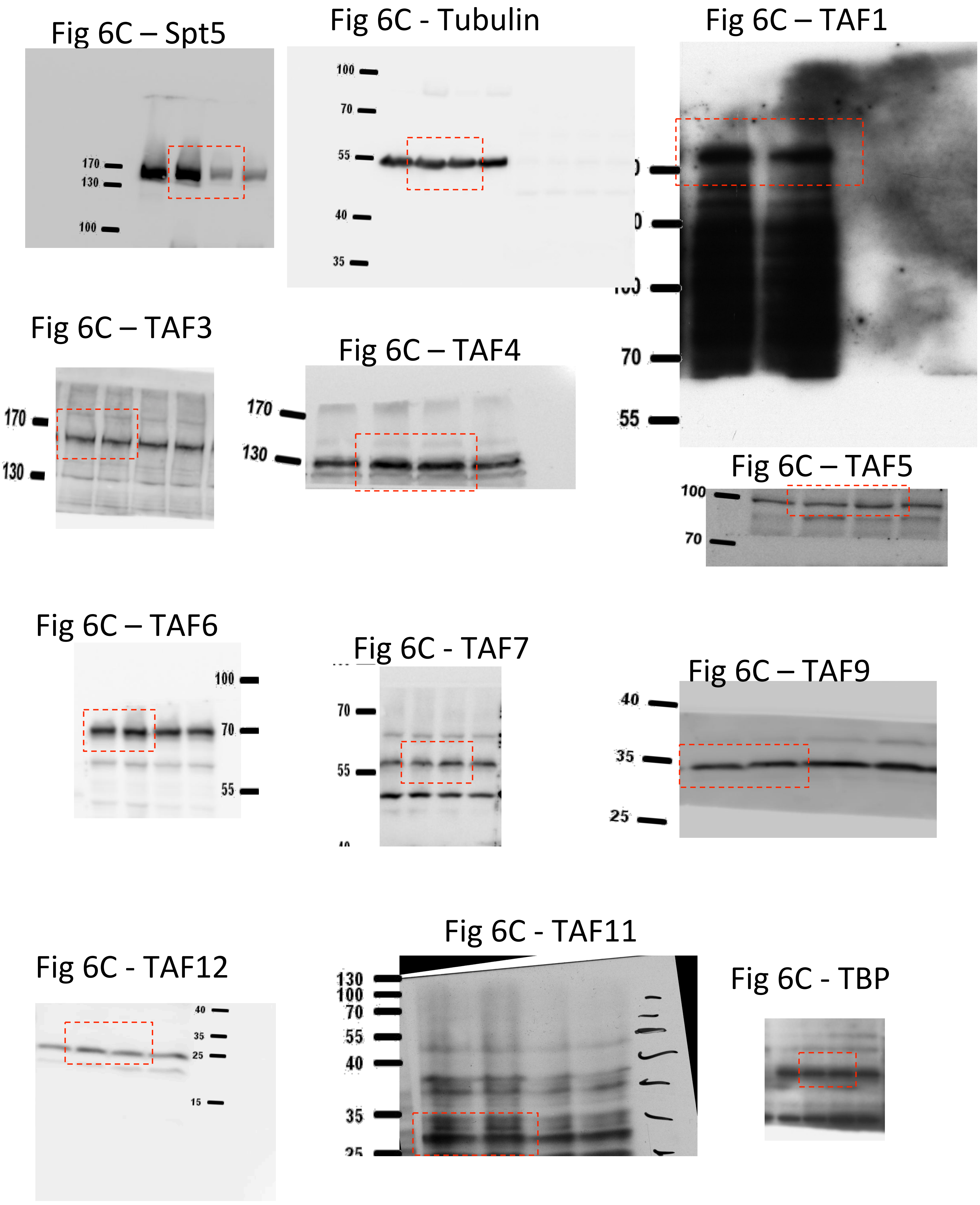

Supplementary Figure 6: Original images of western blots used for preparing figure 6.

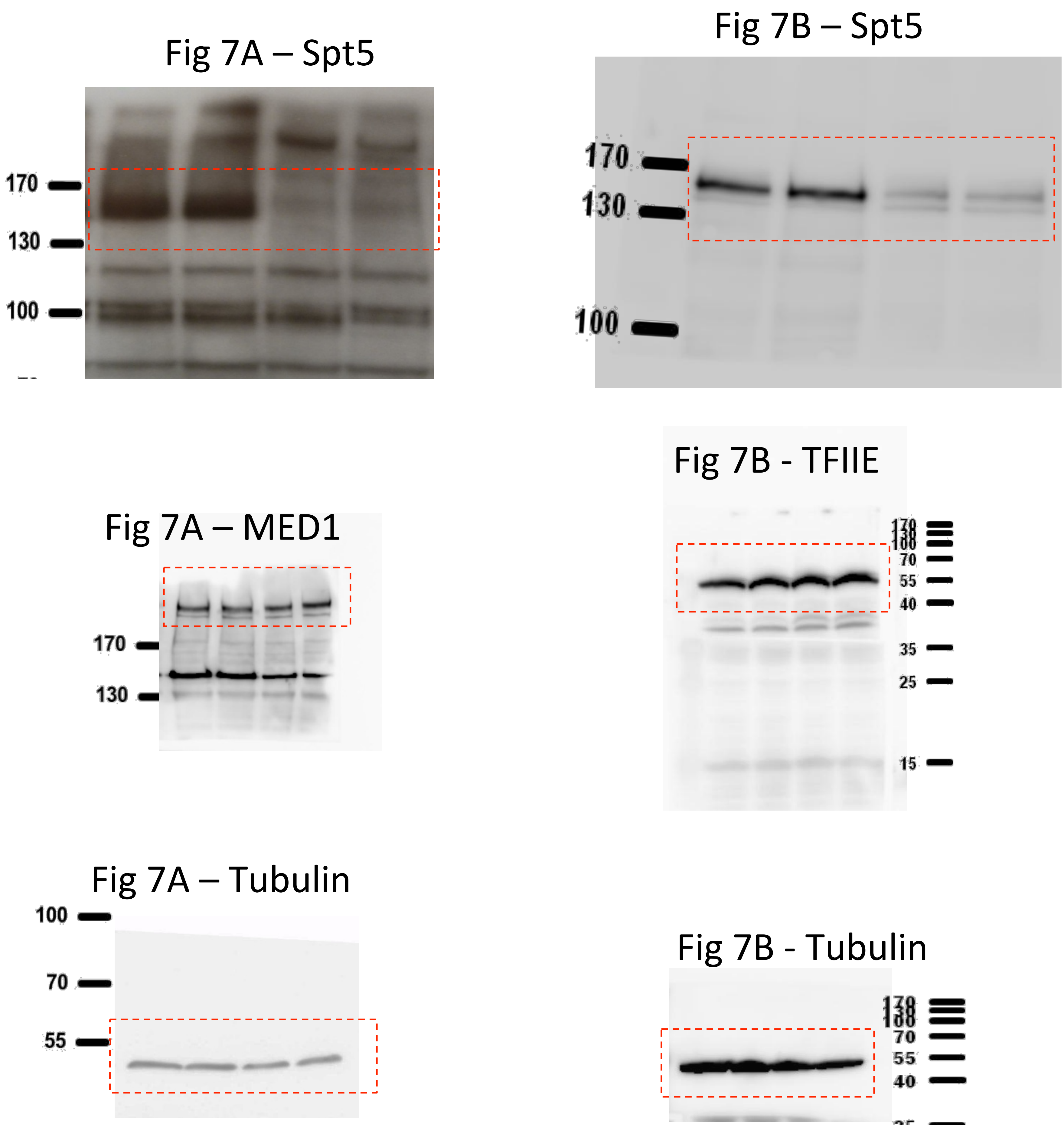

Supplementary Figure 7: Original images of western blots used for preparing figure 7.
